# Structural and Functional Sensing of Bio-Tissues Based on Compressive Sensing Spectral Domain Optical Coherence Tomography

**DOI:** 10.3390/s19194208

**Published:** 2019-09-27

**Authors:** Luying Yi, Xiangyu Guo, Liqun Sun, Bo Hou

**Affiliations:** State Key Laboratory of Precision Measurement Technology & Instruments, Department of Precision Instrument, Tsinghua University, Beijing 100084, China; yily15@mails.tsinghua.edu.cn (L.Y.); guoxy18@mails.tsinghua.edu.cn (X.G.); houb15@mails.tsinghua.edu.cn (B.H.)

**Keywords:** optical coherence tomography, compressive sensing, structural imaging, functional sensing

## Abstract

In this paper, a full depth 2D CS-SDOCT approach is proposed, which combines two-dimensional (2D) compressive sensing spectral-domain optical coherence tomography (CS-SDOCT) and dispersion encoding (ED) technologies, and its applications in structural imaging and functional sensing of bio-tissues are studied. Specifically, by introducing a large dispersion mismatch between the reference arm and sample arm in SD-OCT system, the reconstruction of the under-sampled A-scan data and the removal of the conjugated images can be achieved simultaneously by only two iterations. The under-sampled B-scan data is then reconstructed using the classic CS reconstruction algorithm. For a 5 mm × 3.2 mm fish-eye image, the conjugated image was reduced by 31.4 dB using 50% × 50% sampled data (250 depth scans and 480 spectral sampling points per depth scan), and all A-scan data was reconstructed in only 1.2 s. In addition, we analyze the application performance of the CS-SDOCT in functional sensing of locally homogeneous tissue. Simulation and experimental results show that this method can correctly reconstruct the extinction coefficient spectrum under reasonable iteration times. When 8 iterations were used to reconstruct the A-scan data in the imaging experiment of fisheye, the extinction coefficient spectrum calculated using 50% × 50% data was approximately consistent with that obtained with 100% data.

## 1. Introduction

Spectral-domain optical coherence tomography (SD-OCT) is a rapidly advancing optical frequency-domain imaging modality, which provides not only non-invasive high-resolution three-dimensional (3D) images of biological tissue [1,2,3,4], but also quantification of chromophores in tissues [5,6,7]. Imaging speed and imaging depth are two significant performance indicators of SD-OCT for imaging bio-tissues, especially in vivo imaging and functional activity measurements. However, the improvement of the two indicators comes at a cost: an expensive, large arrays, high speed camera with a high sampling rate. Since the imaging speed and imaging depth of the SD-OCT are mainly determined by the acquisition speed and the spectral resolution of the spectrometer, respectively. In addition, transferring and processing large amounts of data is also challenging [8]. In order to overcome these problems, the compressive sensing SD-OCT (CS-SDOCT) technique was proposed [9,10,11,12,13], and it has been demonstrated that a high-fidelity OCT image can be reconstructed using only 37.5% of *k*-domain spectral data [9]. To further compress the data volume, 2D and 3D CS-SDOCT are investigated successively, in which under-sampling is performed in two or three directions of spectral data [14,15]. In most existing 2D or 3D CS-SDCT methods, the CS algorithm is applied to each dimension in turn [14,15], and the reconstruction time of each dimension is tens of seconds to several minutes [16] (depending on the size of the OCT image), which is very time-consuming. 

In order to reduce the data processing time, and to remove the conjugate images (another important factor affecting the imaging depth) [17,18], we propose to add the dispersion coding (DE) method to the recovery of under-sampled A-scan data in 2D CS-SDOCT in this work. DE is an effective technique for removing conjugated images present in standard SD-OCT systems [19,20,21,22]. Compared to other methods of removing conjugate images, such as phase shifting methods [17,23], modulation methods [24,25], etc., DE requires only one measurement and has inherent phase stability [19,20,21,22]. The combination of DE and 1D CS-SDOCT has been described in our previous work [26], where under-sampling was only implemented in the A-scan, which results in the data not being completely compressed. In this work, we combine the DE method with the 2D CS-SDOCT to reconstruct a full depth OCT image from the 2D under-sampled spectral data. By introducing a large dispersion mismatch between the reference arm and the sample arm in the SD-OCT system, reconstruction of the under-sampled A-scan data and removal of the conjugate images can be simultaneously performed through only two iterations. Hundreds of under-sampled depth scans are typically reconstructed in 1–2 s, which greatly improves reconstruction efficiency compared to tens of seconds or even minutes used in existing 2D CS-SDOCT models.

Most existing studies are investigating the application of CS in the imaging of biological tissue structures, but little research has been done on functional sensing. In many practical applications, we hope to obtain both the structural image and the atlas of the functional parameters, such as the blood oxygen parameters of the tissue calculated from the extinction coefficient spectrum [7,27]. Therefore, in this paper, we preliminarily analyze the influence of full-depth 2D CS-SDOCT method on the quantification of extinction coefficient spectrum.

The remainder of this paper is organized as follows. In Section 2, we introduce the basic principles of structural imaging and functional sensing for SD-OCT, the data processing steps for the full depth 2D CS-SDOCT model, and the experimental configurations used in this article. Section 3 presents the experimental results of fisheye structure imaging, and Section 4 summarizes the simulation results and experimental results of functional sensing. Finally, in Section 5 we provide a brief conclusion.

## 2. Methods

### 2.1. Structural and Functional Sensing Based on SD-OCT

In SD-OCT, when the DC signal and noise are removed, the interference signal at the lateral position *x* can be expressed as [22]:

(1) $$I(x,k)=S(k)R[\int_{0}^{\infty }a(x,z)\exp(i2kz)\exp(i\varphi (k))dz+\int_{0}^{\infty }a*(x,z)\exp(-i2kz)\exp(-i\varphi (k))dz],$$

where *S*(*k*) is the power spectrum function of the light source; *R* is the amplitude of the reflected light from the reference arm; *a* (*x*, *z*) is the amplitude of scattered light from the sample at depth *z*; *k* is the wave number; and phase φ(k) is introduced due to dispersion in order to remove conjugated image. After compensating for dispersion, we can obtain:

(2) $$T(x,k)=I(x,k)\exp(-i\varphi (k))=S(k)R[\int_{0}^{\infty }a(x,z)\exp(i2kz)dz+\int_{0}^{\infty }a*(x,z)\exp(-i2kz)\exp(-i2\varphi (k))dz].$$

After implementing the inverse fast Fourier transform (*IFFT*) on Equation (2), the conjugate signal a*(*x*, *z*) broadened due to exponential factor exp(−i2φ(k)) can be removed by DE method, and the real structure signal can be obtained:

(3) H(x,z)=IFFT[T(x,k)]=IFFT[S(k)]⊗[Ra(x,z)].

Considering the attenuation of light intensity, Equation (3) becomes [7]:

(4) $$H(x,z)=IFFT\left[S(k)\right]⊗\left[R\alpha \mu_{b}(x,z)\exp(-2z\mu_{t}(x,z))\right],$$

in which *μ_t_* and *μ_b_* are the extinction coefficient and the backscattering coefficient, respectively, α is a constant, and $a(x,z)=\alpha \mu_{b}(x,z)$. By replacing the *IFFT* in Equation (3) with a short-time Fourier transform (*STFT*), we can obtain a depth-resolved spectrum *H* (*k*, *z*):

(5) $$H(x,k,z)=STFT\left[S(k)\right]⊗\left[R\alpha \mu_{b}(x,k,z)\exp(-2z\mu_{t}(x,k,z))\right].$$

The extinction coefficient of the tissue consists of an absorption coefficient and a scattering coefficient, which reflects most of the functional activities of the tissue. Therefore, many functional parameters of the tissue can be measured after the extinction coefficient spectrum $\mu_{t}(x,k,z)$ is obtained.

Equations (4) and (5) describe the intensity expression in homogeneous medium, or in a homogeneous depth range within the medium, which can be determined by pre-segmenting the medium [28]. In this case, the extinction coefficient and the backscattering coefficient are considered to be constant over this local depth range. Therefore, the extinction coefficient may now be estimated by [29]:

(6) $$\mu_{t}(x,k,z)=-\frac{1}{D}\ln\left(\frac{H(x,k,z_{1})}{H(x,k,z_{2})}\right),$$

where *D* is the optical path difference of this depth range, and *z*_1_ and *z*_2_ are the starting and ending coordinates of this depth range, respectively. To reduce the sensitivity to speckle noise, this method is typically replaced by fitting an exponential function to the data over a certain depth range [30].

### 2.2. Full-Depth 2D CS-SDOCT

**Figure 1:**
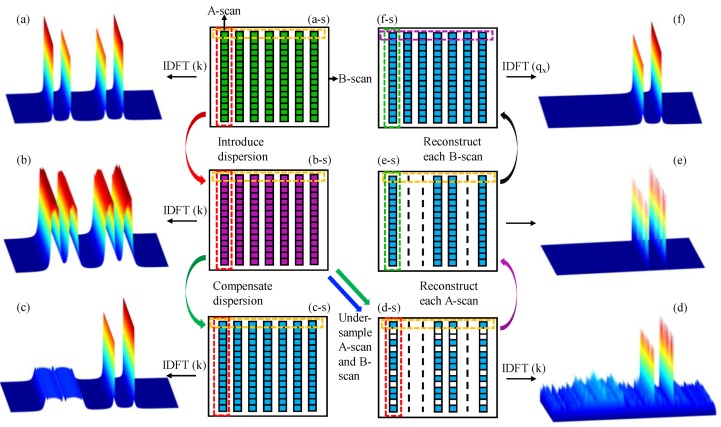
Schematic demonstration of the reconstruction strategy. (**a**–**f**) the patterns of the spectral data matrixes; (**a-s**)–(**f-s**) the simulation results of two mirror surfaces.

The principle and reconstruction process of full depth 2D CS-SDOCT are illustrated vividly by Figure 1. 

In Figure 1, the middle two columns are patterns of the spectrum ***I*** (*x*, *k*), and the left and right columns are reconstructed 2D structural images of the two mirror surfaces P_1_ and P_2_. To explain, Equation (1) can be formulated as a digital signal as follows [26]:

(7) I(x,k)=2S(k)RRe[ΦΨa(x,z)],

where ***a*** represents the full-depth structural signals in the spatial domain; Ψ represents the discrete Fourier transform; and Φ represents the dispersive measurement matrix. The pattern of **I** (*x*, *k*) is shown in Figure 1b-s, and the structure image obtained by performing inverse discrete Fourier transform (*IDFT*) on its *k*-domain is shown in Figure 1b, in which both the real signals and the conjugate signals are broadened. Figure 1a,a-s show the structural image and spectrum pattern without dispersion.

After under-sampling the *x*-domain and *k*-domain of the spectral matrix ***I*** (*x*, *k*), we can get 2D under-sampled spectral data:

(8) I(xc,kc)=2S(k)RRe[MxMkΦΨa(x,z)],

in which **M**_x_ and **M**_k_ is the sampling masks in *x*-domain and *k*-domain, respectively, and the subscript *c* indicates that the parameter is under-sampled. The pattern of ***I*** (*x_c_*, *k_c_*) is shown in Figure 1d-s, and the structure image obtained by performing *IDFT* on its *k*-domain is shown in Figure 1d, where the dispersion has been compensated. We can find that real structure signals are enhanced and not broadened. On the contrary, the conjugate signals are broadened and suppressed. Figure 1c is the structural image reconstructed from the complete spectral matrix shown in Figure 1c-s that has been compensated for dispersion. Compared to Figure 1c, there are many un-acquired points in the lateral spatial domain in Figure 1d, and the axial spatial domain of Figure 1d has conjugate signals as well as incoherent aliasing artifacts caused by under-sampling in *k*-domain of spectral data.

Each A-scan is first reconstructed, that is, *a* (*x*_c_, *z*) is obtained first. Equation (7) can be expressed as:

(9) I(xc,kc)=2S(k)RRe[MkΦΨa(xc,z)].

We define the measurement matrix ${Τ}_{k}=M_{k}\Phi \Psi$, and CS reconstructs $a(x_{c},z)$ by solving the following constrained optimization problem:

(10) mina||a(xc,z)||1 s.t.||2Re[Tka(xc,z)]−I(xc,kc)||22≤ε,

in which *ε* is the parameter that controls the fidelity of the signal in the measurement domain; ${\left\|•\right\|}_{1}$ and ${\left\|•\right\|}_{2}$ are the *l*_1_ norm and *l*_2_ norm, respectively. The two-step compressive DE (TCDE) method is used to solve this problem [26].

In the TCDE method, a large dispersion mismatch is introduced between the reference arm and sample arm to achieve strong suppression of mirror image artifacts, and two iterations are performed to reconstruct the full-depth OCT image from under-sampled spectral data. The first iteration selects the signal peaks with higher intensity and the signals in the previous and next pixels of these peaks, followed by the removal of their conjugate items and incoherent aliasing artifacts caused by under-sampling. The second iteration selects the signals with lower intensity. 

Finally, the complete spectral matrix and spatial structure signals of each A-scan are reconstructed, as shown in Figure 1e-s,1e, respectively. We can find that the conjugate images and incoherent artifacts present in *z* direction are removed.

Each B-scan is then reconstructed, that is, *a* (*x*, *z*) is obtained. After the reconstruction of A-scan, Equation (7) now becomes:

(11) I(xc,z)=2S(k)RRe[Mxa(x,z)].

We define the measurement matrix ${Τ}_{x}=M_{x}\Psi^{H}$, where $\Psi^{H}$ is the *IDFT* operator, and CS reconstructs $f(q_{x},z)=\Psi a(x,z)$ by solving the following constrained optimization problem:

(12) minf||f||1 s.t.||2Re(Txf)−I(xc,z)||22≤ε.

*q*_x_ is the spatial frequency of *x*. After the **f** is reconstructed, the signal *a* (*x*, *z*) can be obtained as $a(x,z)=\Psi^{H}f$. The reconstructed complete spectral matrix and structure image are shown in Figure 1f-s and Figure 1f, respectively. The reason why *a* (*x*, *z*) is not directly recovered is that the measurement domain cannot be the same as the sparse domain [14], so we first transform the signal *a* (*x*, *z*) to a sparser domain for reconstruction.

As of now, we not only obtain the complete structure signal a(x,z), but also obtain the corresponding complete spectral data I(x,k), where no conjugate term and dispersion exist. After the *STFT* is applied to the *k*-domain of I(x,k), the depth-resolved spectrum H(x,k,z) expressed in Equation (5) can be obtained.

Through the above steps, a full depth 2D structural image and the spectrum of *μ*_t_ are reconstructed from the 2D compressed data.

### 2.3. Hardware and Software Setup

**Figure 2:**
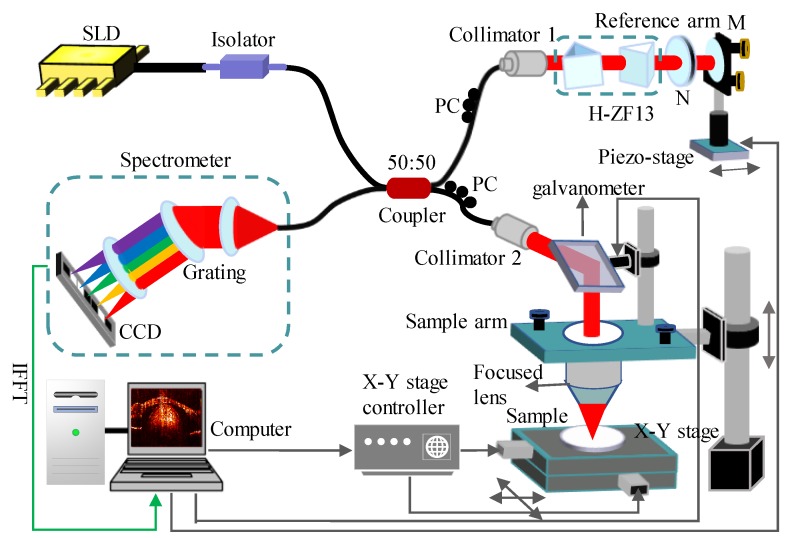
Schematic diagram of the SD-OCT system. PC: polarization controller, M: mirror, and N: neutral density filter.

Figure 2 shows the schematic diagram of the optical setup for the SD-OCT used in this study. A Super-luminescent diode (SLD-331, Superlum Ltd, Russia) with a center wavelength of 790 nm and a full width at half maximum (FWHM) of 45 nm was utilized. The light emitted from the SLD was divided into two equal light beams using a 50:50 fiber coupler (TW805R5F2, Thorlabs, Newton, NJ, USA), which were sample light and reference light, and then both beams were transformed into collimated spatial light by the collimators. The sample was scanned in the *x*-direction using a one-dimension galvanometer scanner (TSH8310, Sunny technology, Beijing, China) to obtain a 2D image, and the rotating axis of the scanning mirror was at the focus of the focusing lens to form a telecentric optical path. The sample arm had an electrically controlled moving platform, which was used to adjust the samples to an appropriate position for imaging. A pair of prisms (H-ZF13) was placed in the reference arm to increase the dispersion mismatch of the system, in order to remove the conjugate image by the DE method. The reference arm’s power was controlled by a neutral density filter. The piezo stage was used to achieve phase shifting so as to remove fundamental frequency noise. The polarization of the two light beams was controlled by the polarization controller. The backscattered/reflected light from the sample and reference light reflected from the reference mirror interfered at the optical fiber coupler, which were detected by a spectrometer (Maya 2000 pro, Ocean Optics) with a spectral resolution of 0.05 nm. The focal length of the focusing lens used in the system was 30 mm. The theoretical axial and lateral resolutions of the SD-OCT system in air were approximately 6.88 μm and 7.54 μm, respectively. Further details about the system configuration can be found in Table 1.

The trigger of each step in the system was controlled by a LabVIEW-based host computer program. The raw spectral data acquired from the spectrometer was transferred to a computer, and then was processed according to the steps described in Section 2.2. Finally, a full depth 2D structural image and the extinction coefficient spectrum $\mu_{t}(x,k,z)$ of the sample were obtained. 

## 3. Structural Imaging Results

**Figure 3:**
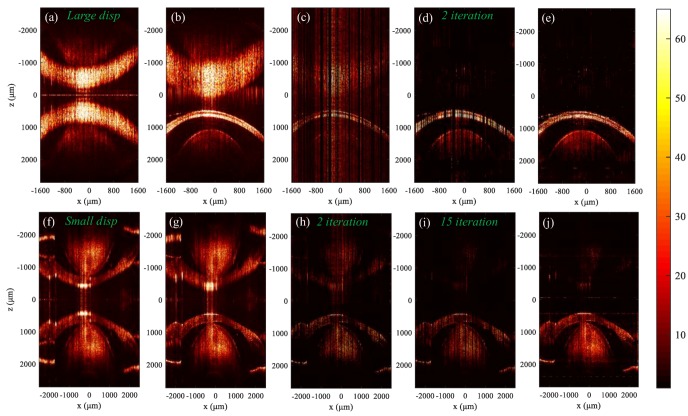
Structural imaging results using a fisheye. (**a–e**) show the results of the system with large dispersion mismatch, and (**i**–**j**) show the results for the system with small dispersion. (**a**,**f**) Images without dispersion compensation; (**b**,**g**) Images with dispersion compensation; (**c**) Image obtained from 2D under-sampled spectral data; (**d**,**h**,**i**) Results after reconstruction of axial data; (**e**) and (**j**) Reconstructed full-depth 2D images.

The feasibility of the data processing method introduced in Section 2.2 was investigated based on the results for a fisheye, as shown in Figure 3.

During the experiment, the goldfish was kept alive by wrapping in wet facial tissue (except for the eye). Drops of fresh water were applied to the fisheye every minute to prevent it from dehydrating. Figure 3a–e show the cross-sectional images of the fish eye obtained by the experimental system with large dispersion mismatch, and Figure 3a–e correspond to Figure 1b–f shown in Section 2.2, respectively. The dispersion coefficients were [22]: $a_{2}=10492\times 10^{-30}s^{2},a_{3}=376\times 10^{-45}s^{2}$. Figure 3a,b are the images reconstructed from complete spectral data before and after dispersion compensation, respectively. We can see that both the conjugate image and the real image in Figure 3a are broadened due to the dispersion mismatch. The broadening of the real image in Figure 3b is removed and the conjugate term is further broadened and suppressed. Figure 3c is a structural image reconstructed from 2D spectral data that is under-sampled at a sampling rate of 50% × 50%. Figure 3d is the image after reconstruction of all selected A-scan data using the TCDE method, which indicates that the conjugate image and the axial incoherent aliasing artifacts are removed. It took 1.2 s to reconstruct all selected A-scan data. Figure 3e shows the full depth 2D structural image in which both lateral and axial data were reconstructed, and the mirror image artifact was reduced by 31.4 dB, which demonstrates the feasibility of the data processing method described in Section 2.2 in applications to in vivo imaging. It should be noted that the use of TCDE was in the process of reconstructing A-scan data, while the SpaRSA algorithm was used for the reconstruction of B-scan data [15,16], and all B-scan data were reconstructed in 105 s. Therefore, we removed the conjugate image and reduced the time spent on the existing 2D CS-SDOCT [15] by almost half.

Figure 3f–j show the images of the fish eye obtained by the experimental system with smaller dispersion mismatch. The dispersion coefficients were: $a_{2}=2500\times 10^{-30}s^{2},a_{3}=795\times 10^{-45}s^{2}$. Figure 3f,g are the images reconstructed using complete spectral data before and after dispersion compensation, respectively. Figure 3h is the image after reconstruction of its axial data using the TCDE method, where the conjugate image was not well eliminated, and only 9 dB was reduced. Accordingly, the dispersion mismatch of the system should be large enough to remove conjugate image by less iterations. Otherwise, we can only increase the number of iterations to get a high-fidelity OCT image. Figure 3i,j are the results after 15 iterations, and the conjugate image was reduced by 26.6 dB in Figure 3j. 

## 4. Functional Sensing Results

### 4.1. Simulation Results

**Figure 4:**
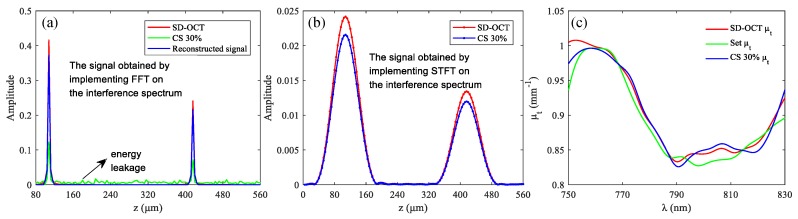
Simulation results of functional sensing. (**a**) The signals obtained by *IFFT* processing of spectral data; (**b**) the signals obtained by *STFT* processing of spectral data; and (**c**) extinction coefficient spectrum $\mu_{t}(k)$.

We started by generating a spatial domain SD-OCT A-scan with 2000 data points, and each pixel represented a depth of 4 μm. Two reflectors R_1_ and R_2_ were introduced in the A-scan: ***a*** (27) = 1 and ***a*** (104) = 1, representing the walls of a blood vessel, as shown in Figure 4a. Accordingly, the diameter of the blood vessel was 308 μm. The positions of R_1_ and R_2_ as well as the diameter were arbitrarily set. The power spectrum function *S*(*k*) of the light source defined in Equation (1) was a gaussian window with the full width at half maximum (FWHM) of 80 nm, and the central wavelength was 790 nm. The spacing between the two reflectors was simulated as a homogeneous blood vessel, and the set extinction coefficient spectrum of blood is shown in green curve of Figure 4c [31]. The detected interference spectrum can be expressed as [29]:

(13) I(k)=2Re{S(k)0.5μt(k)[exp(i2k⋅108)+exp(−2μt(k)⋅308)exp(i2k⋅416)]},

where the $\mu_{b}(k)$ was set to be half of the $\mu_{t}(k)$ [30]. We think this setting does not affect the investigation results of the effect of the CS algorithm on functional sensing.

The results in Figure 4a were obtained by implementing *IFFT* on the interference spectrum expressed in the Equation (13). The red and green curves in Figure 4a were obtained from the complete spectral data and 30% spectral data, respectively, and the blue curve represents the structural signal reconstructed using the CS reconstruction algorithm. We implemented a *STFT* on Equation (13) to obtain the depth-resolved spectrum *H* (*k*, *z*), and the *STFT* function used in the simulation was the built-in ’spectrogram’ function in MATLAB software. The window width used in the *STFT* was 100 data points. The results are shown in Figure 4b, where the red and blue curves were obtained from the complete spectral data and 30% spectral data. We can see that the structural signals in Figure 4b are broadened compared to those in Figure 4a, i.e., the spatial resolution is degraded. This is because the spatial resolution and the spectral resolution in the *H* (*k*, *z*) obtained using *STFT* are contradictory. The curves in Figure 4c are the extinction coefficient spectrum calculated according to Equation (6), and the red and blue curves were calculated from complete spectral data and 30% spectral data.

**Figure 5:**
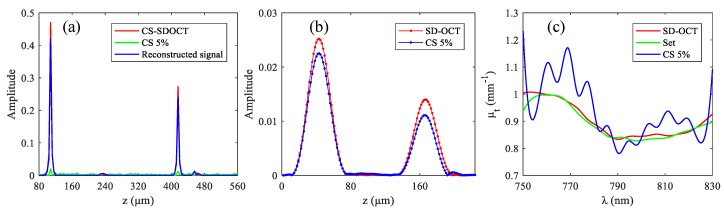
Simulation results of functional sensing sampling rate is 5%. (**a**) The signals obtained by *IFFT* processing of spectral data; (**b**) the signals obtained by *STFT* processing of spectral data; and (**c**) spectrum of extinction coefficient $\mu_{t}(k)$.

The simulation results show that the use of CS has little influence on the functional sensing of SD-OCT. This is because although under-sampling results in energy leakage of the signal, the ratio of leaked energy to the actual value of each signal is approximately the same. That is, in Figure 4b, the ratio of the two peaks in the red curve is the same as that in the blue curve, but there is still a slight difference between the two. In Figure 4b, the ratios are 1.8016 and 1.8012, respectively. The reason for this slight difference is that the signal at each depth position is the sum of the actual signal and the incoherent artifacts of other signals at that position. While the difference is not significant when the sampling rate is not particularly low because the artifacts are relatively small compared to the actual signal, but when the sampling rate gets smaller, the difference may become larger, as shown in Figure 5, in which the sampling rate is 5%. 

At the sampling rate of 5%, the structural signal still seems to be recovered well. However, due to the large energy leakage ratio seen in the green curve of Figure 5a, the ratio of the two peaks in the blue curve of Figure 5b differs greatly from that in the red curve. In Figure 5b, the ratios are 1.8016 and 2.0288, respectively. Therefore, the calculated extinction coefficient was incorrect, as shown by the blue curve in Figure 5c.

In summary, when the sampling rate is not particularly low, the use of CS does not theoretically affect the correct measurement of functional parameters of the tissue.

### 4.2. Experimental Results

**Figure 6:**
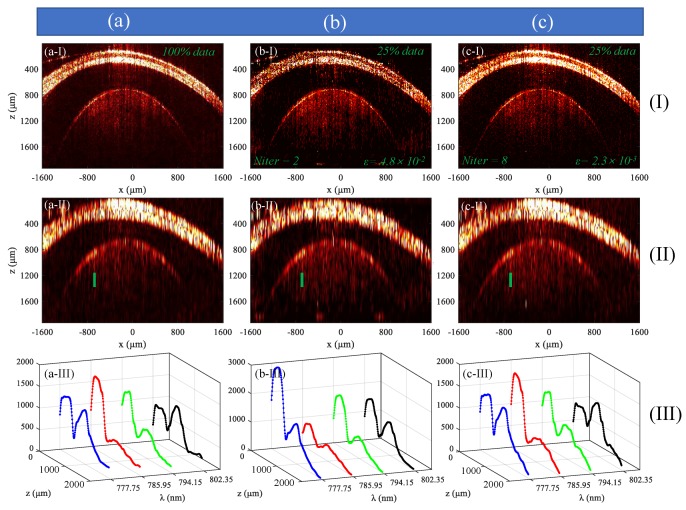
Experiment results of functional sensing. The results in (**a**) were obtained using 100% data. (**b**) and (**c**) are the results after 2 iterations and 8 iterations using 50% × 50% data. (**I**) The structural image obtained by implementing *IFFT* on spectral data; (**II**) the structural image obtained by implementing *STFT* on spectral data; (**III**) depth-resolved spectrum *H* (*k*, 0:2000 μm). For clarity, only the curves of four wavelengths were plotted in (**III**).

**Figure 7:**
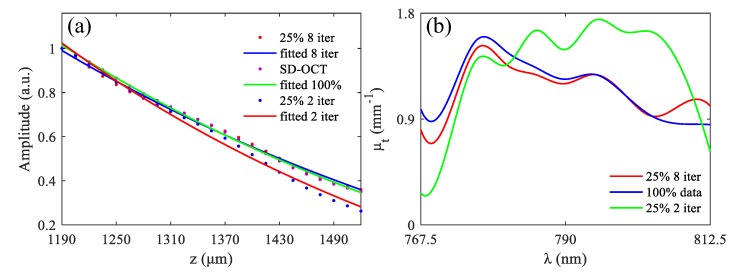
(**a**) The results of exponential fitting; (**b**) spectrums of the extinction coefficient.

The spectral data of the fisheye shown in Figure 3 was used to analyze the impact of CS on functional sensing of SD-OCT, and the results are shown in Figure 6 and Figure 7.

Figure 6a–c represent maps of the first column, the second column, and the third column, respectively; I, II, and III represent maps of the first row, the second row, and the third row, respectively. Pictures in Figure 6I are 2D structure images obtained by *IFFT* processing of spectral data, and the results in Figure 6II, III represent structural images and depth-resolved spectrums obtained by *STFT* processing of spectral data, respectively. In the *STFT*, the wavelength resolution was 3 nm. The results in Figure 6a got from the complete spectral data. Figure 6b,c show the results after 2 iterations and 8 iterations using 50% × 50% data, and the corresponding residual signals *ε* defined in Equation (12) were 4.8 × 10^−2^ and 2.3 × 10^−3^, respectively. We made the conjugate rejection ratio of both cases about 30 dB by reasonably selecting the preset light intensity threshold for each iteration.

The lens of the fisheye was considered to be a homogeneous medium here, so we can use Equation (6) to calculate the extinction coefficient spectrum. The depth range of 1190 μm–1520 μm at the lateral position *x* = 940 μm indicated by the green short lines in Figure II was randomly selected for exponential fitting. Figure 7a shows the results of exponential fitting of function *H* (790 nm, 1190 μm–1520 μm) in the three cases mentioned in Figure 6, and the results in Figure 7b are the extinction coefficient spectrum $\mu_{t}(k)$ in the three cases. It can be seen from the results in Figure 7 that the extinction coefficient spectrum reconstructed using 2 iterations was significantly different from that reconstructed with 100% data, while the spectrum reconstructed using 8 iterations was approximately consistent with that obtained with 100% data. This is because in the result of the 2 iterations, many small structure signals were removed in order to suppress more conjugate signals. The time for 8 iterations was 5 s, which is still much less than tens of seconds or even minutes used in classical CS algorithm.

Consequently, in 2D CS-SDOCT, we need to set the number of iterations reasonably, which is equivalent to setting the value of ε, to ensure that the removed signals are noise rather than small structural signals, which can be achieved by calibrating the system before conducting imaging.

Next, several issues need to be explained.

First, the performance of the full depth 2D CS-SDOCT system needs to be discussed. In the system shown in Figure 2, a pair of dispersive prisms was placed in the reference arm so that it did not affect the penetration depth of the light beam in sample. Although large dispersion mismatch results in reduced intensity and axial resolution of the detected signal due to spectrometer limitations [19], the dispersion mismatch introduced herein is approximately equal to that at *d* = 20 mm in [19], Therefore, the dispersion mismatch introduced in this work has little effect on the detection sensitivity and axial resolution of the signal. Another point to note is that the dispersion compensation method used in this paper is based on the assumption that the dispersion of the sample itself is homogeneous, so not axial resolutions of all depth were corrected to the theoretical limit. Recently, a study using numerical intensity correlation method to achieve all-depth dispersion cancellation was reported [32], and 2D CS technique is also applicable to this method.

Secondly, the simulations and experiments on functional sensing conducted in this section are all to demonstrate that the extinction coefficient spectrum can be consistent with that obtained from 100% data despite the use of CS. We believe this is an important basis for exploring the potential application of CS-SDOCT in functional sensing. The extinction coefficient spectrum obtained using 100% data is not necessarily accurate due to the presence of noise. It has been demonstrated that the use of CS can improve the SNR of OCT image [11], so we predict that the value of ε can be reasonably set so that the recovered extinction coefficient spectrum is more accurate than that obtained using 100% data. We will discuss this issue quantitatively in future work.

Thirdly, the tissues discussed in this paper were all locally homogeneous, such as the vascular model used in the simulation and the fisheye used in the experiment. We will explore the potential application of full depth 2D CS-SDOCT in more complex tissues, where extinction coefficient spectrum in a smaller depth range or even a resolution depth range needs to be obtained. We can predict that the requirements for reconstruction accuracy will be higher in this case. 

Finally, although the most state-of-the-art SD-OCT systems are very fast, CS makes significant sense for dynamic measurements of large-sized tissues.

## 5. Conclusions

In this paper, we studied the application of compressive sensing SD-OCT (CS-SDOCT) in the structural imaging and functional sensing for bio-tissues. We combined 2D CS technology with dispersion encoding method to reconstruct 2D full-depth fish-eye structure images using 25% spectral data. In the case where large dispersion mismatching was introduced into system, the conjugate image can be mostly suppressed after only two iterations. For a 5 mm × 3.2 mm fish-eye image, the conjugated image was reduced by 31.4 dB using 50% × 50% sampled data (250 depth scans and 480 spectral sampling points per depth scan), and all A-scan data was reconstructed in only 1.2 s. Furthermore, we used simulations and experiments to analyze the influence of the use of CS on functional sensing, and concluded that the reconstructed extinction coefficient spectrum can be consistent with that obtained from 100% data despite the use of CS. 

## Figures and Tables

**Table 1:** The parameters of the SD-OCT system.

System Parameters	Specification
Lateral resolution	7.54 μm
Axial resolution (air/tissue)	6.88 μm/4.74 μm
Optical power of light source	24 mW
Maximum imaging depth (air/tissue)	3.12 × 2 mm/2.15 × 2 mm
Maximum imaging width	21.8 mm
Working distance of sample arm	22mm
